# RIPK3-Mediated Necroptosis in Diabetic Cardiomyopathy Requires CaMKII Activation

**DOI:** 10.1155/2021/6617816

**Published:** 2021-06-07

**Authors:** Yun Chen, Xinshuai Li, Yuyun Hua, Yue Ding, Guoliang Meng, Wei Zhang

**Affiliations:** ^1^Department of Pharmacology, School of Pharmacy, Nantong University, Key Laboratory of Inflammation and Molecular Drug Target of Jiangsu Province, Nantong, 226001 Jiangsu, China; ^2^School of Medicine, Nantong University, Nantong, China

## Abstract

Activation of Ca^2+^/calmodulin-dependent protein kinase (CaMKII) has been proved to play a vital role in cardiovascular diseases. Receptor-interaction protein kinase 3- (RIPK3-) mediated necroptosis has crucially participated in cardiac dysfunction. The study is aimed at investigating the effect as well as the mechanism of CaMKII activation and necroptosis on diabetic cardiomyopathy (DCM). Wild-type (WT) and the RIPK3 gene knockout (RIPK3^−/−^) mice were intraperitoneally injected with 60 mg/kg/d streptozotocin (STZ) for 5 consecutive days. After 12 w of feeding, 100 *μ*L recombinant adenovirus solution carrying inhibitor 1 of protein phosphatase 1 (I1PP1) gene was injected into the caudal vein of mice. Echocardiography, myocardial injury, CaMKII activity, necroptosis, RIPK1 expression, mixed lineage kinase domain-like protein (MLKL) phosphorylation, and mitochondrial ultrastructure were measured. The results showed that cardiac dysfunction, CaMKII activation, and necroptosis were aggravated in streptozotocin- (STZ-) stimulated mice, as well as in (Lepr) KO/KO (db/db) mice. RIPK3 deficiency alleviated cardiac dysfunction, CaMKII activation, and necroptosis in DCM. Furthermore, I1PP1 overexpression reversed cardiac dysfunction, myocardial injury and necroptosis augment, and CaMKII activity enhancement in WT mice with DCM but not in RIPK3^−/−^ mice with DCM. The present study demonstrated that CaMKII activation and necroptosis augment in DCM via a RIPK3-dependent manner, which may provide therapeutic strategies for DCM.

## 1. Introduction

Diabetes mellitus (DM) is an independent risk factor of diabetic cardiomyopathy (DCM), in the deficiency of coronary artery diseases, hypertension, and other cardiovascular risk factors. DCM is characterized by a series of structural and functional abnormalities, including myocardial stiffness, contractility impairment, myocardial fibrosis, and hypertrophy [1, 2]. Special attention must be paid to DCM due to its concealed onset, rapid evolution but poor treatment efficacy. Possible pathophysiological factors of DCM involve hyperglycemia, hyperlipidemia, insulin resistance, oxidative stress, endoplasmic reticulum stress, cardiomyocyte death, and mitochondrial dysfunction [3, 4]. However, the exact mechanism is still obscure. Investigation to find out potential preventative and therapeutic strategies is an urgent problem to be solved.

Recent advances have revealed a new necrotic type of regulated cell death, necroptosis, which is morphologically distinct from apoptosis and necrosis [5]. Necroptosis is characterized by cell enlargement, organelle swelling, and plasma membrane rupture, followed by cell disintegration and intracellular component release without obvious changes in nuclear chromatin [6]. Receptor-interaction protein kinase 3 (RIPK3) plays the core role in the necroptotic signaling pathway. RIPK3, along with receptor-interacting protein 1 (RIP1) and mixed lineage kinase domain-like protein (MLKL), executes necroptosis [7, 8]. Besides, Zhang et al. identified Ca^2+^/calmodulin-dependent protein kinase (CaMKII) as a novel substrate of RIPK3 mediating ischemia- and oxidative stress-induced myocardial necroptosis [9]. Emerging evidences have corroborated the vital role of necroptosis in cardiovascular diseases. However, contribution of necroptosis in DCM remains insufficient.

CaMKII is a serine/threonine kinase which has various functions including regulation of key proteins involved in Ca^2+^ handling, intercellular coupling, cell death, inflammation, and mitochondrial function [10–13]. CaMKII can be activated by binding to calcium-bound calmodulin (Ca^2+^/CaM), autophosphorylation, oxidation, *S*-nitrosylation, and *O*-GlcNAcylation [14–16]. Activation of CaMKII mediates physiological or pathological responses and remodeling under cardiac stresses. It has been well established that CaMKII plays a prominent role in myocardial hypertrophy, pressure overload-induced cardiac hypertrophy and fibrosis, ischemia/reperfusion (I/R) injury, heart failure (HF), postmyocardial infarction (MI) remodeling, and ventricular arrhythmias [15, 17–21]. Recently, some studies have revealed the potential contribution of CaMKII in DCM [10]. Therefore, identification of the mechanism of CaMKII is beneficial to aid in providing a novel pharmacologic target for DCM.

CaMKII has four isoforms: *α*, *β*, *γ*, and *δ*, among which CaMKII*δ* is prominent in the heart. CaMKII*δ* expresses three variants CaMKII*δ*A, CaMKII*δ*B, and CaMKII*δ*C after alternative splicing of its exon 14, 15, or 16 by splicing factors. Alternative splicing of CaMKII*δ* is strictly regulated. Once it is disordered, the expression of three variants imbalances to lead to cardiomyocyte dysfunction and ultimately heart diseases [22].

Protein phosphatase 1 (PP1) is a serine/threonine phosphatase mainly present in the heart and plays a vital role in regulating the phosphorylation of splicing factors to eventually mediate CaMKII*δ* alternative splicing [14]. PP1 also regulates CaMKII dephosphorylation. It has been shown that PP1 expression was elevated in the heart of heart failure patients [23]. However, the clinical application value of PP1 has not been found. Inhibitor 1 of protein phosphatase 1 (I1PP1) binds to PP1 and subsequently inhibits its activity. I1PP1 transgenic mice demonstrated minor hypertrophy after transverse aortic constriction (TAC). Our previous studies have demonstrated that CaMKII regulation by I1PP1 alleviates necroptosis in high glucose-induced cardiomyocyte injury [24].

In the present study, we investigated the role of necroptosis in the development of DCM and the molecular mechanism underlying necroptosis. We also try to demonstrate the contribution of I1PP1 on the regulation of CaMKII*δ* alternative splicing and CaMKII activity to ameliorate necroptosis in the myocardium of streptozotocin- (STZ-) induced wild-type and RIPK3^−/−^ diabetic mice and db/db mice.

## 2. Materials and Methods

### 2.1. Animals

Male 8-week C57BL/6 mice (wild type, WT) were provided by the Experimental Animal Center of Nantong University (Nantong, China). RIPK3 knockout (RIPK3^−/−^) mice with C57BL/6 background were donated by the Institute of Molecular Medicine, Peking University (Beijing, China).

Male 12-week (Lepr) KO/KO mice (db/db) and (Lepr) WT/WT mice purchased from GemPharmatech Limited Company (Nanjing, China) were fed in separate cages. All the procedures were in accordance with both the recommendations of the Guidelines for the Care and Use of Laboratory Animals published by the National Institutes of Health and the Instructional Animal Care and Use Committee of Nantong University (approval no. NTU-20161225).

### 2.2. Establishment of Mouse Diabetic Models

After adaptive feeding for a week, mice were injected with 60 mg/kg/d STZ (Sigma, USA), dissolved in 0.1 mol/L citrate buffer for 5 consecutive days after a 12-hour overnight fast [25]. Mice in the control group were injected with the same amount of citrate buffer. Mice with fasting blood glucose (FBG) level above 16.7 mmol/L were considered as the diabetic mice. Mice were randomly divided into different groups: WT group (WT), WT mice with DCM group (WT-DCM), RIPK3 KO group (RIPK3^−/−^), and RIPK3 KO mice with DCM group (RIPK3^−/−^-DCM).

### 2.3. Measurement of the Glycosylated Hemoglobin (HbA1c)

12 weeks after STZ injection, blood samples were collected and heparinized. The level of HbA1c was measured using the Glycosylated Hemoglobin A1c Assay Kit according to the manufacturer's instructions (Jiancheng Bioengineering Institute, Nanjing, China).

### 2.4. Measurement of Triglyceride (TG)

Blood samples were obtained and centrifuged at 1000 g for 10 min. The supernatant was collected, and serum TG content was measured with the Triglyceride Assay Kit (Jiancheng Bioengineering Institute, Nanjing, China). Absorbance was recorded at the wavelength of 510 nm.

### 2.5. Echocardiography

12 weeks after STZ injection, the mice were anesthetized with 1.5% isoflurane and examined by 2-D guided M-mode echocardiography (Visual Sonic Vevo 2100, Toronto, ON, Canada) to assess cardiac configuration and function. Ejection fraction (EF) was calculated with the percentage of the difference between end-diastolic volume (EDV) and end-systolic volume (ESV) to EDV, whereas fractional shortening (FS) was calculated with the percentage of the difference between end-diastolic diameter (EDD) and end-systolic diameter (ESD) to EDD. In addition, the ratio of early (E) to late (A) diastolic velocity ratio (E/A) was calculated. All indexes were obtained from three consecutive beats.

### 2.6. I1PP1 Adenovirus Injection

Recombinant adenovirus solution carrying I1PP1 gene (100 *μ*L) was injected into the caudal vein of mice as previously mentioned. Mice in the control group were injected with recombinant adenovirus solution carrying GFP gene (100 *μ*L) from the caudal vein. The efficiency and effect of I1PP1 adenovirus injection were assessed by western blot for further study.

### 2.7. Hematoxylin-Eosin (HE) Staining

The left ventricles were fixed with 4% paraformaldehyde overnight, embedded in paraffin, and then cut into sections at 5 *μ*m thickness. The sections were stained with hematoxylin and eosin successively and dehydrated with ethanol. Then, sections were observed and photographed under an optical microscope.

### 2.8. Observation of Myocardial Ultrastructure

Hearts were cut into pieces of 1 mm^3^ and fixed with 4% glutaraldehyde for 2 h at 4°C and 1% osmium tetroxide for 2 h at room temperature successively. The samples were dehydrated, infiltrated, embedded with Epon812, and cut into ultrathin sections of 60-80 nm and then stained with uranyl acetate and lead citrate. Myocardial ultrastructure was examined with a transmission electron microscope (TEM) (HT7700, HITACHI, Japan), and the number of mitochondria was counted.

### 2.9. Cardiac Troponin I (cTnI) Measurement

12 weeks after STZ injection, the myocardium was cut up, homogenated, and centrifuged at 2000 rpm for 20 min. The supernatants were collected and incubated with mouse Cardiac Troponin I ELISA Kit (Jiancheng Bioengineering Institute, Nanjing, China). Absorbance was recorded at the wavelength of 450 nm.

### 2.10. TdT-Mediated dUTP Nick End Labeling (TUNEL) Staining

The left ventricles were fixed with 4% paraformaldehyde overnight, embedded in paraffin, and then cut into sections at 5 *μ*m thickness. After staining with TUNEL (Beyotime, Shanghai, China) at 37°C for 60 min, the sections were rinsed 3 times with PBS and then observed and photographed under an optical microscope and quantified using Image J analysis software.

### 2.11. Real-Time Polymerase Chain Reaction

The total RNA of the myocardium was extracted from the myocardium using a Trizol separation reagent. The cDNA was then synthesized by the Prime Script™ RT Master Mix Kit (Takara, Kyoto, Japan) before quantitative real-time PCR were performed using the SYBR Green Fast qPCR mix (Takara) with the ABI StepOne PCR System (ABI, Carlsbad, CA, USA). All primers used were the following: atrial natriuretic peptide- (ANP-) F 5′-GAGAAGATGCCGGTAGAAGA-3′, ANP-R 5′-AAGCACTGCCGTCTCTCAGA-3′; brain natriuretic peptide (BNP-F), 5′-CTGCTGGAGCTGATAAGAGA-3′, BNP-R 5′-TGCCCAAAGCAGCTTGAGAT-3′; CaMKII*δ*A-F 5′-CGAGAAATTTTTCAGCAGCC-3′, CaMKII*δ*A-R 5′-ACAGTAGTTTGGGGCTCCAG-3′; CaMKII*δ*B-F 5′-CGAGAAATTTTTCAGCAGCC-3′, CaMKII*δ*B-R 5′-GCTCTCAGTTGACTCCATCATC-3′; CaMKII*δ*C-F 5′-CGAGAAATTTTTCAGCAGCC-3, CaMKII*δ*C-R 5′-CTCAGTTGACTCCTTTACCCC-3′; 18S-F 5′-AGTCCCTGCCCTTTGTACACA-3′, 18S-R 5′-CGATCCGAGGGCCTCACTA-3′. Experimental cycle threshold values were normalized to 18S, a housekeeping gene, and relative mRNA expression was calculated versus a control sample.

### 2.12. Western Blot

The same amount of protein sample extraction from the myocardium was separated with sodium dodecyl sulfate-polyacrylamide gel electrophoresis (SDS-PAGE) and transferred to polyvinylidene difluoride (PVDF) membrane (Millipore, Massachusetts, USA). The membrane was blocked with 5% skim milk dissolved in TBST for 1 h at room temperature and then incubated with primary at 4°C overnight. The primary antibodies used were listed as follows: anti-I1PP1 (1 : 1000) and anti-PP1 (1 : 1000) (Santa Cruz Biotechnology, Santa Cruz, CA, USA); anti-ox-CaMKII (1 : 1000) (Millipore, Kenilworth, NJ, USA); anti-CaMKII (1 : 1000) (Abcam, Cambridge, UK); anti-caspase 3, anti-cleaved caspase 3, anti-MLKL, anti-p-MLKL, and anti-RIPK1 (1 : 1000, Cell Signaling Technology, Danvers, MA, USA); anti-p-CaMKII (1 : 1000, Thermo Fisher Scientific, Rockford, IL, USA); anti-GAPDH (1 : 5000, Sigma-Aldrich, St. Louis, MO, USA). Horseradish peroxidase- (HRP-) conjugated secondary antibodies were incubated for 1.5 h at room temperature. Protein bands were visualized with enhanced chemiluminescence (ECL) (Thermo Fisher Scientific Inc., Rockford, IL, USA). The target protein expression level was normalized to the level of GAPDH.

### 2.13. Statistical Analysis

All data were presented as standard error of the mean (S.E.M.). Statistical analysis was performed by the unpaired Student *t* test on comparisons between two groups and by the one-way ANOVA test and followed by the Bonferroni post hoc test on comparisons among multiple groups. *P* < 0.05 was considered statistically significant.

## 3. Results

### 3.1. Cardiac Dysfunction, CaMKII*δ* Activity, and Necroptosis Are Augmented in DCM

**Figure 1:**
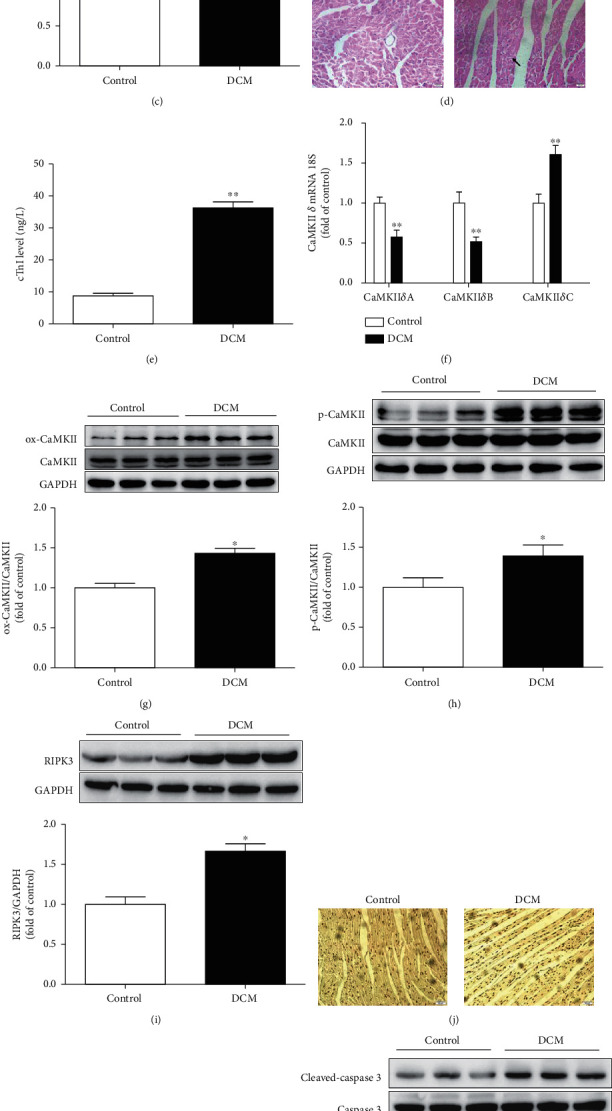
Cardiac dysfunction, CaMKII*δ* activity, and necroptosis are augmented in DCM. Male C57BL/6 mice were injected with 60 mg/kg/d STZ for 5 consecutive days after a 12-hour overnight fast. Mice in the control group were injected with the same amount of citrate buffer. (a–c) Cardiac function was assessed by echocardiography, and EF, FS, and E/A were calculated. (d) Myocardium injury was measured by HE staining. Bar = 20 *μ*m. (e) cTnI was detected. (f) The mRNA levels of CaMKII*δ*A, CaMKII*δ*B, and CaMKII*δ*C of the myocardium were detected by quantitative real-time PCR. 18S was serviced as a housekeeping mRNA. (g–h) Expression of CaMKII oxidation (ox-CaMKII), CaMKII phosphorylation (p-CaMKII), and total CaMKII was quantified by western blot. GAPDH was used as a loading control. (i) RIPK3 protein expression was quantified by western blot. GAPDH was used as a loading control. (j, k) Cell apoptosis of myocardium was detected with TUNEL staining and quantified with Image J analysis software. Bar = 50 *μ*m. (l) Cleaved-caspase 3 and caspase 3 protein expression were quantified by western blot. GAPDH was used as a loading control. ^∗^*P* < 0.05 and ^∗∗^*P* < 0.01, significantly from control, *n* = 6.

FPG, HbA1c, serum TG, and myocardial hypertrophic gene expression were increased significantly in mice with DCM, which suggested that STZ injection produced diabetes mellitus (Figure S1). Mice with FBG more than 16.7 mmol/L were confirmed as diabetic mice and used for further experiments. Compared to the mice in the control group, mice with DCM suffered diastolic and systolic dysfunctions, elucidated by decreased EF, FS, and E/A (Figures 1(a)–1(c)). It suggested that DCM was successfully established in the present study. HE staining displayed hypertrophy, distortion, and irregular arrangement of myocardial cells in the hearts of mice with DCM (Figure 1(d)). The cTnI level was detected to evaluate myocardial injury. The cTnI level was significantly higher in the DCM group than that in the control group (Figure 1(e)). Disorder of CaMKII*δ* alternative splicing is prone to promote cardiomyocyte dysfunction and ultimately heart diseases [24, 26]. Since no specific antibodies of CaMKII*δ* variants were available, CaMKII*δ*A, CaMKII*δ*B, and CaMKII*δ*C mRNA expressions were measured by quantitative real-time PCR. There was a significant decrease of CaMKII*δ*A and CaMKII*δ*B but an increase of CaMKII*δ*C in diabetic mice, indicating disorder of CaMKII*δ* alternative splicing in mice with DCM (Figure 1(f)). Moreover, oxidation and phosphorylation of CaMKII also increased in the DCM group (Figures 1(g) and 1(h)). Various studies have confirmed that RIPK3 was involved in necroptosis. Our study verified that RIPK3 was significantly increased in the hearts of DCM mice (Figure 1(i)). As apoptosis is a critical manifestation of necroptosis, TUNEL staining and cleaved-caspase 3 expression indicated that the necroptotic cardiomyocytes in mice with DCM were significantly more than that in the control mice (Figures 1(j)–1(l)). All these data suggested that cardiac dysfunction, CaMKII*δ* activity, and necroptosis were augmented in DCM.

**Figure 2:**
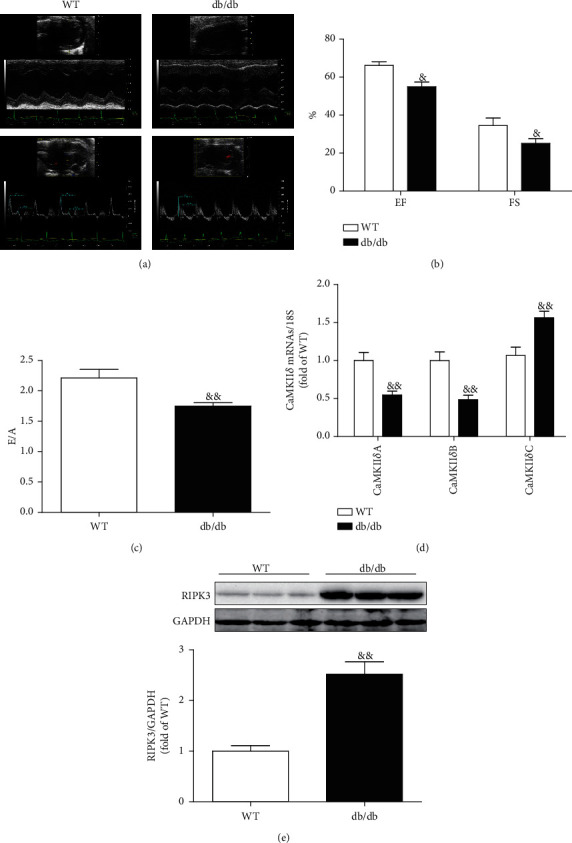
Cardiac dysfunction, CaMKII*δ* activity, and RIPK3 expression are augmented in db/db mice. (a–c) In db/db and its wild-type mice, cardiac function was assessed by echocardiography and EF, FS, and E/A were calculated. (d) In db/db and its wild-type mice, the mRNA levels of CaMKII*δ*A, CaMKII*δ*B, and CaMKII*δ*C of the myocardium were detected by quantitative real-time PCR. 18S was serviced as a housekeeping mRNA. (e) In db/db and its wild-type mice, RIPK3 protein expression was quantified by western blot. GAPDH was used as a loading control. *^&^P* < 0.05 and ^&*&*^*P* < 0.01, significantly from WT. *n* = 6.

In accordance, both systolic and diastolic cardiac functions of db/db mice were significantly impaired as compared to WT mice (Figures 2(a)–2(c)), CaMKII*δ* variant expression disordered and RIPK3 expression was increased in the myocardium of db/db mice (Figures 2(d) and 2(e)).

### 3.2. RIPK3 Deficiency Alleviates Cardiac Dysfunction, CaMKII*δ* Alternative Splicing Disorder, and Necroptosis in DCM

**Figure 3:**
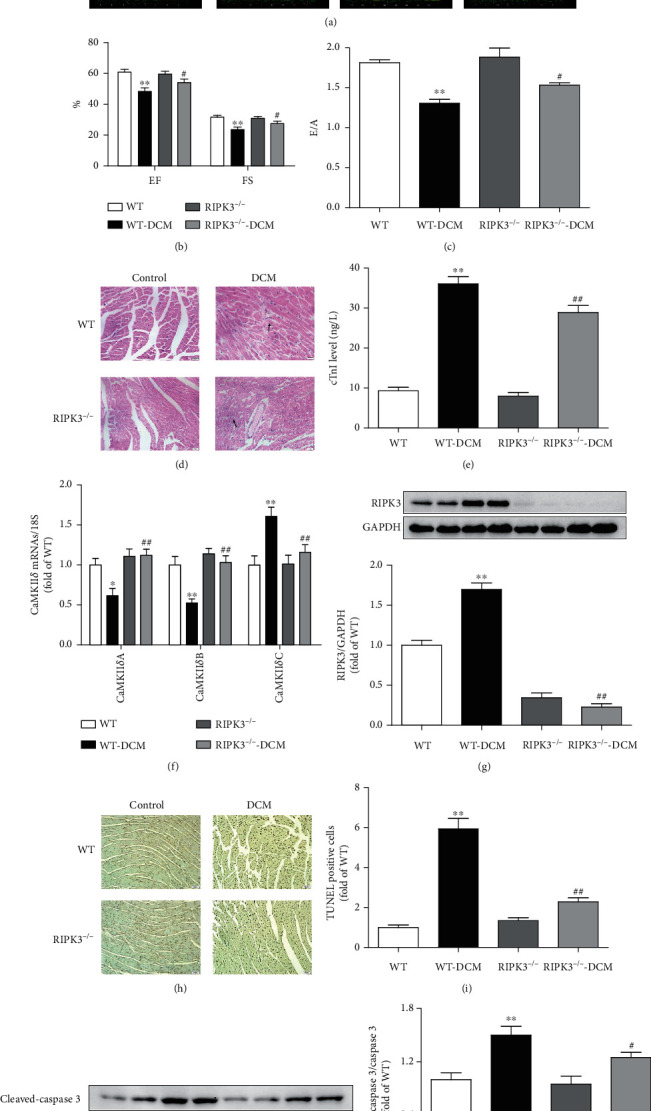
RIPK3 deficiency alleviates cardiac dysfunction, CaMKII*δ* alternative splicing disorder, and necroptosis in DCM. Male C57BL/6 mice and RIPK3 knockout mice (RIPK3^−/−^) were injected with 60 mg/kg/d STZ for 5 consecutive days after a 12-hour overnight fast. WT and RIPK3^−/−^ mice in the control group were injected with the same amount of citrate buffer. (a–c) Cardiac function was assessed by echocardiography, and EF, FS, and E/A were calculated. (d) Myocardium injury was measured by HE staining. Bar = 20 *μ*m. (e) cTnI was detected. (f) The mRNA levels of CaMKII*δ*A, CaMKII*δ*B, and CaMKII*δ*C of the myocardium were detected by quantitative real-time PCR. 18S was serviced as a housekeeping mRNA. (g) RIPK3 expression was quantified by western blot. GAPDH was used as a loading control. (h, i) Cell apoptosis of the myocardium was detected with TUNEL staining. (j, k) Cleaved-caspase 3 and caspase 3 protein expression were quantified by western blot. GAPDH was used as a loading control. ^∗∗^*P* < 0.01 and ^∗^*P* < 0.05 significantly from WT; ^##^*P* < 0.01 and #*P* < 0.05 significantly from WT-DCM. *n* = 6.

To further investigate the contribution of RIPK3 in the development of DCM, RIPK3-KO (RIPK3^−/−^) mice were used in our study. In diabetic mice, RIPK3 deficiency significantly enhanced EF, FS, and E/A, suggesting both systolic and diastolic functions were improved (Figures 3(a)–3(c)). Notably, RIPK3^−/−^ hearts were resistant to myocardial injury in DCM, as evidenced by improved structure of myocardium and reduced cTnI level (Figures 3(d) and 3(e)). Disorder of CaMKII*δ* alternative splicing was alleviated in RIPK3 deficiency mice with DCM (Figure 3(f)). TUNEL staining and western blot assay revealed that necroptosis was attenuated in RIPK3 deficiency mice with DCM (Figures 3(g)–3(k)). These data suggested that the impairment of cardiac dysfunction and the augment of necroptosis were attenuated in mice with DCM if RIPK3 was deficient.

### 3.3. RIPK3 Deficiency Decreases RIPK1 Expression, MLKL Phosphorylation, and CaMKII Activity and Improves Myocardial Mitochondrial Ultrastructure in Mice with DCM

**Figure 4:**
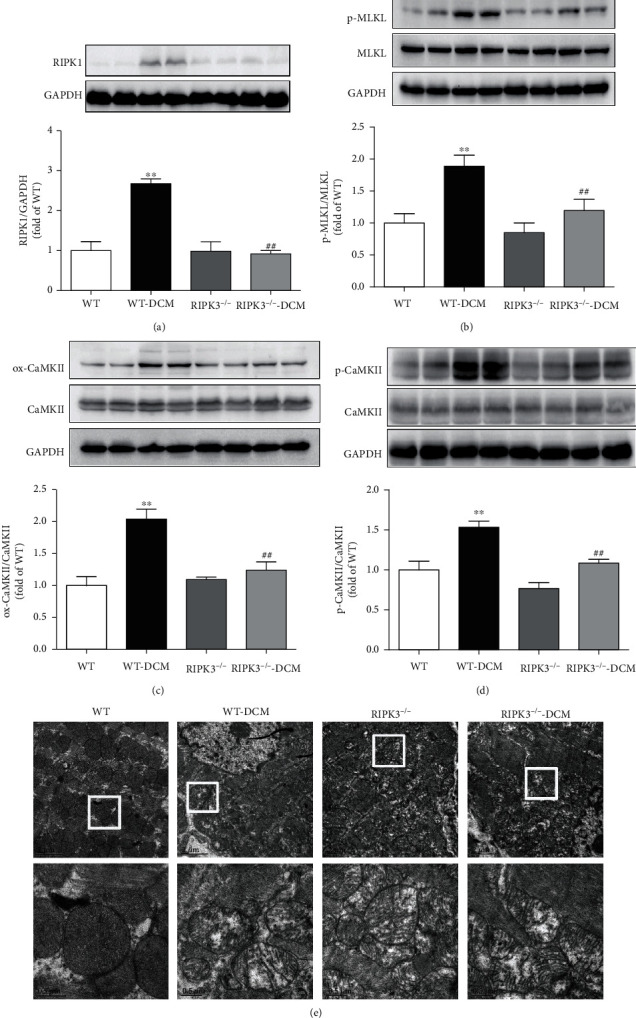
RIPK3 deficiency decreases RIPK1 expression, MLKL phosphorylation, and CaMKII activity and improves myocardial mitochondrial ultrastructure in DCM. (a) RIPK1 expression in cardiomyocytes was quantified by western blot. GAPDH was used as a loading control. (b) MLKL phosphorylation (p-MLKL) and total MLKL were quantified by western blot. GAPDH was used as a loading control. (c, d) Expression of CaMKII oxidation (ox-CaMKII), CaMKII phosphorylation (p-CaMKII), and total CaMKII was quantified by western blot. GAPDH was used as a loading control. (e) Myocardial mitochondrial ultrastructure was examined with a transmission electron microscope. Scale bars: 2 *μ*m (upper) and 0.5 *μ*m (lower), respectively. ^∗∗^*P* < 0.01, significantly from WT; ^##^*P* < 0.01 and #*P* < 0.05, significantly from WT-DCM. *n* = 6.

Besides RIPK3, previous studies have demonstrated that RIPK1 also participated in the process of necroptosis, and phosphorylation of MLKL was an essential effector molecule of necroptosis [27]. In the present study, RIPK1 expression and MLKL phosphorylation were markedly elevated in the myocardium of mice with DCM, which were significantly decreased in RIPK3^−/−^ mice with DCM (Figures 4(a) and 4(b)). A recent study identified CaMKII as one of the substrates of RIPK3 in cardiac ischemic disease [8]. Our results revealed that RIPK3 deficiency attenuated CaMKII oxidation and phosphorylation in the myocardium of mice with DCM (Figures 4(c) and 4(d)). TEM analysis of myocardial mitochondrial ultrastructure showed irregular and swelled mitochondria and fractured mitochondrial cristae in the left ventricular myocardium of mice with DCM. However, the abnormalities of mitochondria with DCM were improved in RIPK3^−/−^ mice (Figure 4(e)).

### 3.4. I1PP1 Overexpression Reverses Cardiac Dysfunction, Myocardial Injury, and Necroptosis Augment in Mice with DCM

**Figure 5:**
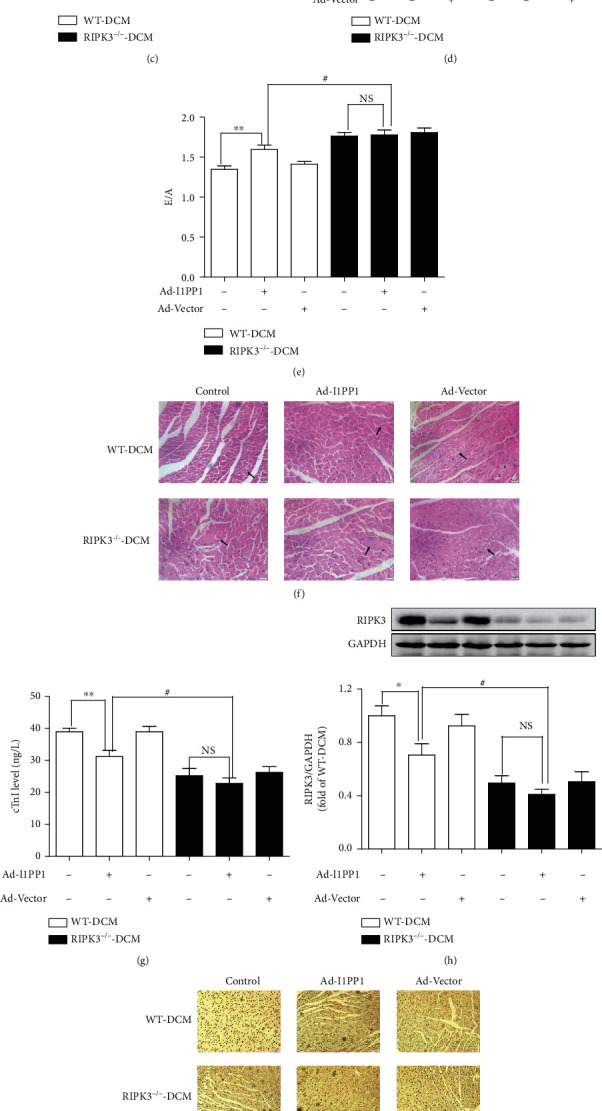
I1PP1 overexpression reverses cardiac dysfunction, myocardial injury, and necroptosis augment in mice with DCM. 100 *μ*L recombinant adenovirus solution carrying I1PP1 gene or vector was injected into the caudal vein of mice 12 weeks after STZ injection or injected into the caudal vein of db/db mice or its WT mice. (a) The timeline of animal treatment was summarized. (b–e) Cardiac function was assessed by echocardiography, and EF, FS, and E/A were calculated. (f) Myocardium injury was measured by HE staining. Bar = 20 *μ*m. (g) cTnI was detected. (h) RIPK3 expression was quantified by western blot. GAPDH was used as a loading control. (i) Cell apoptosis of the myocardium was detected with TUNEL staining. (j) Cleaved-caspase 3 and caspase 3 protein expression were quantified by western blot. GAPDH was used as a loading control. ^∗∗^*P* < 0.01 and ^∗^*P* < 0.05; ^##^*P* < 0.01 and #*P* < 0.05; NS: no significance, *n* = 6.

To further evaluate the relative contribution of CaMKII*δ* variant disorder in DCM, the recombinant adenovirus carrying the I1PP1 gene was directly injected into the caudal vein of mice to regulate CaMKII*δ* alternative splicing. Two weeks after injection, western blot assay showed that I1PP1 was overexpressed and significantly inhibited PP1 expression in the myocardium of mice (Figure S2). EF, FS, and E/A were improved after I1PP1 overexpression in WT mice with DCM but not in RIPK3^−/−^ mice with DCM. Moreover, cardiac function of RIPK3^−/−^ mice with DCM was further improved as compared to WT mice with DCM after I1PP1 overexpression (Figures 5(a)–5(e)). Similarly, I1PP1 overexpression reduced cTnI level, improved myocardial structure, and alleviated necroptosis augment in WT mice with DCM but not in RIPK3^−/−^ mice with DCM. In addition, lower cTnI level, better myocardial structure, and weaker necroptosis augment were detected in RIPK3^−/−^ mice with DCM as compared to WT mice with DCM after I1PP1 overexpression (Figures 5(f)–5(j)). All the above data suggested CaMKII was possible downstream of RIPK3 during necroptosis in DCM.

**Figure 6:**
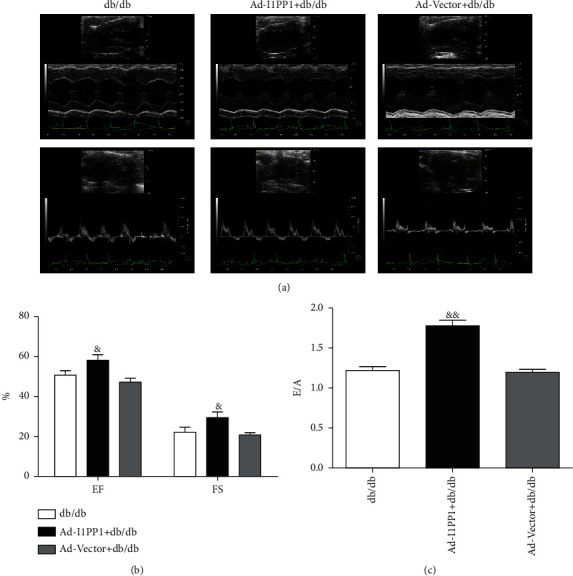
I1PP1 overexpression reverses cardiac dysfunction of db/db mice. (a–c) In db/db and its WT mice, cardiac function was assessed by echocardiography and EF, FS, and E/A were calculated. ^&&^*P* < 0.01 and ^&^*P* < 0.05 significantly from db/db. *n* = 6.

The effects of I1PP1 overexpression were also detected in db/db mice. Results showed that EF, FS, and E/A were significantly elevated in db/db mice after I1PP1 overexpression (Figure 6), indicating CaMKII regulation was beneficial to improve cardiac function of db/db mice.

### 3.5. I1PP1 Overexpression Inhibits Oxidation and Phosphorylation of CaMKII and Improves Myocardial Mitochondrial Ultrastructure in Mice with DCM

**Figure 7:**
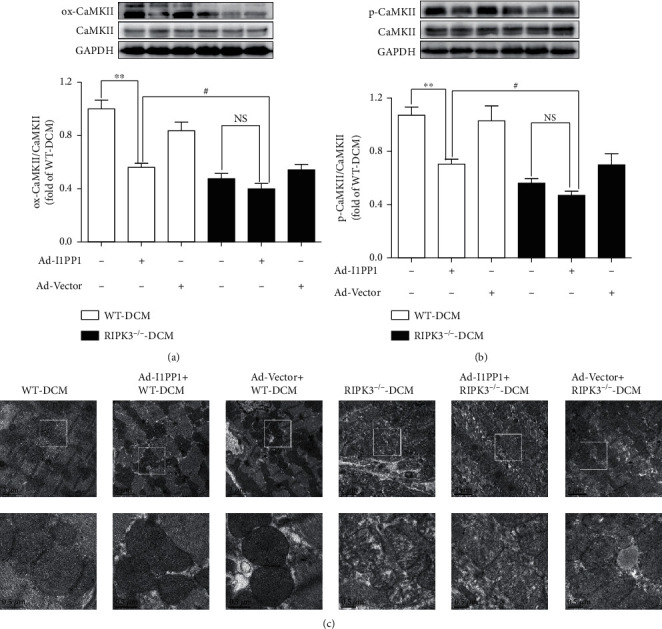
I1PP1 overexpression inhibits oxidation and phosphorylation of CaMKII and improves myocardial mitochondrial ultrastructure in DCM mice. (a, b) Expression of CaMKII oxidation (ox-CaMKII), CaMKII phosphorylation (p-CaMKII), and total CaMKII was quantified by western blot. GAPDH was used as a loading control. (c) Myocardial mitochondrial ultrastructure was examined with a transmission electron microscope. Scale bars: 2 *μ*m (upper) and 0.5 *μ*m (lower), respectively. ^∗∗^*P* < 0.01 and #*P* < 0.05; NS: no significance. *n* = 6.

Further studies were done to investigate the mechanism of I1PP1 overexpression on necroptosis in DCM mice. Oxidation and phosphorylation of CaMKII were inhibited after I1PP1 overexpression in WT mice with DCM but not in RIPK3^−/−^ mice with DCM. Lower CaMKII oxidation and phosphorylation were measured in RIPK3^−/−^ mice with DCM as compared to WT mice with DCM after I1PP1 overexpression (Figures 7(a) and 7(b)). I1PP overexpression improved mitochondrial ultrastructure in WT mice with DCM but not in RIPK3^−/−^ mice with DCM. Better mitochondrial ultrastructure was observed in RIPK3^−/−^ mice with DCM as compared to WT mice with DCM after I1PP1 overexpression (Figure 7(c)).

## 4. Discussion

Accumulated evidence has shown that diabetes results in structural and functional impairment of the hearts. DCM features glucotoxicity, lipotoxicity, and hypertrophy. The results showed increased FBG, HbA1c, serum TG, ANP, and BNP expression in mice with DCM. However, there are still no specific strategies for prevention and treatment of DCM [28]. Necroptosis, a form of regulated necrosis, has been contributable to cardiovascular diseases, such as myocardial infarction, atherosclerosis, and abdominal aortic aneurysm [29]. Our study verified that necroptosis was augmented in mice with DCM. Given RIPK3 is a critical molecule for necroptosis in large amount types of cells, the differences between WT mice and RIPK3^−/−^ mice during DCM were further investigated. Furthermore, RIPK3^−/−^ alleviated cardiac dysfunction, myocardial injury, and necroptosis in diabetic mice.

RIPK1, one common partner of RIPK3, interacts with RIPK3 via a shared RIP homotypic interaction motif domain to form a complex called “necrosome” [30, 31]. Necrostatin-1 (Nec-1), an inhibitor of RIPK1, exerts a protective effect against necroptosis during multiple diseases [32]. MLKL, a recently acknowledged substrate of RIPK3, oligomerizes and translocates to the plasma membrane after being phosphorylated by RIPK3, finally leading to necroptotic cell death. A previous study demonstrated that MLKL deficiency protected against necroptosis in cells including tumor cells, macrophages, and fibroblasts [33–35]. Our results revealed that both RIPK1 expression and MLKL phosphorylation were significantly upregulated in the myocardium of mice with DCM. Moreover, RIPK3^−/−^ mice with DCM showed decreased RIPK1 expression and MLKL phosphorylation. Anyhow, the detailed pathway of RIPK3-evoked myocardial necroptosis was unknown.

CaMKII is a pleiotropic signal that regulates gene expression, contractility, metabolism, Ca^2+^ cycling, and cell survival of cardiomyocytes [36, 37]. CaMKII is inactivated under basal conditions. Sustained activation of CaMKII promotes cardiomyocyte death under conditions of oxidative stress, hyperglycemia, and ischemic and hypoxic injury [38–40]. Zhang et al. reported that RIPK3 contributed to myocardial necroptosis by oxidizing and phosphorylating CaMKII after ischemia-reperfusion injury or doxorubicin challenge [9]. Our previous study demonstrated CaMKII an alternative substrate of RIPK3 in high glucose-induced cardiomyocyte necroptosis [24]. Our present study found significant enhancement of phosphorylation and oxidation of CaMKII both in DCM mice and in AGE-stimulated cardiomyocytes, which were markedly reversed in RIPK3^−/−^ mice. Moreover, no matter I1PP1 is overexpressed or not, cardiac function was improved and necroptosis was suppressed in RIPK3^−/−^ mice. Taken together, the CaMKII signal pathway was possible downstream or substrate of RIPK3 in DCM.

Various studies have confirmed mitochondrial dysfunction as a key underlying mechanism of necroptosis. Mitochondria are a major source of energy and ROS generation, and mitochondrial dysfunction leads to ROS overproduction as well as cell death [41]. RIPK3 increased mitochondrial localization of NADPH oxidase-4 (NOX4) but inhibited mitochondrial complex I and –III and finally promoted kidney tubular injury [42]. Besides, knockdown of RIPK3 reversed the reduced adenosine triphosphate (ATP) production and attenuated the mitochondrial permeability transition pore (mPTP) opening of cardiomyocytes induced by hypoxia-reoxygenation (HR) injury [43]. Our present study found that RIPK3 deficiency improved the ultrastructure of mitochondria in the myocardium of diabetic mice. These results highlighted that mitochondrial injury is possibly responsible for RIPK3-induced necroptosis in DCM.

PP1 is a serine/threonine protein phosphatase that dephosphorylates diverse cellular substrates, acting as a key regulator of some cellular processes [44]. I1PP1 is the first recognized endogenous inhibitor of PP1 and greatly low expressed in cardiomyocytes under basal conditions. Early studies have been reported overexpression of I1PP1 in engineered heart tissue, and rat cardiac myocytes improved contractile function [45]. Our previous studies also uncovered the role of I1PP1 overexpression in protecting against myocardial ischemia-reperfusion injury through CaMKII regulation and necroptosis alleviating in high glucose-induced cardiomyocyte injury [24, 46]. Our present study corroborated the important role of overexpression of I1PP1 in inhibiting necroptosis to alleviate diabetic cardiomyopathy by regulating CaMKII*δ* alternative splicing and CaMKII activity. However, the protective effect of I1PP1 overexpression on DCM was less stronger than that of RIPK3 deficiency. Moreover, I1PP1 overexpression showed no cardioprotective effects in diabetic mice without the RIPK3 gene, indicating that CaMKII might be served as the substrate to mediate necroptosis in the development of DCM.

**Figure 8:**
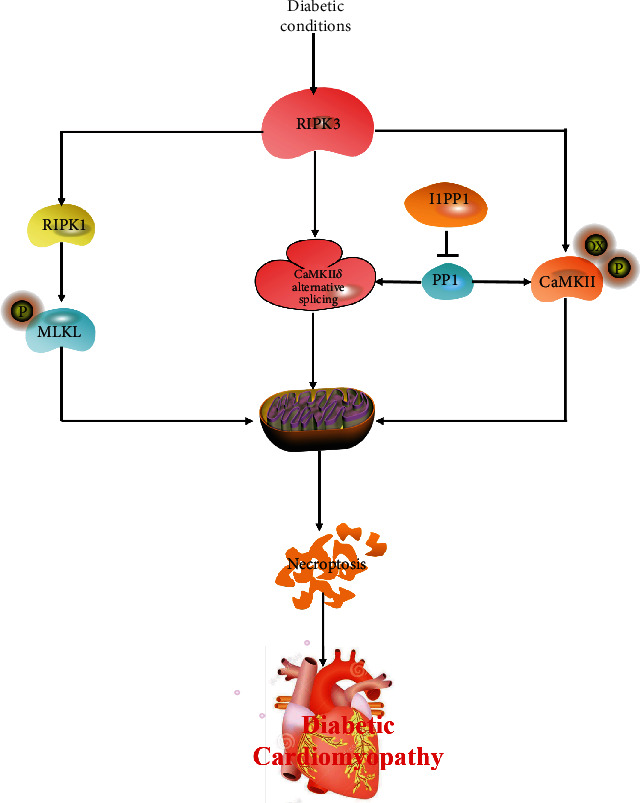
Increased RIPK3 activates RIPK1 expression, MLKL phosphorylation, CaMKII*δ* alternative splicing disorder, oxidation, and phosphorylation of CaMKII under diabetic conditions. I1PP1 overexpression corrects CaMKII*δ* alternative splicing, inhibits CaMKII activation, and attenuates necroptosis in DCM. Taken together, CaMKII activation and necroptosis augment in diabetic cardiomyopathy via RIPK3-dependent manner.

In conclusion, our study proved there was serious necroptosis in DCM. RIPK3 deficiency alleviated myocardial injury, improved cardiac function, suppressed CaMKII activation, and attenuated necroptosis in mice with DCM mice. Taken together, CaMKII activation and necroptosis augment in diabetic cardiomyopathy via a RIPK3-dependent manner (Figure 8). These findings will be a benefit to the understanding of the pathology of diabetic cardiomyopathy and provide novel therapeutic strategies for diabetic cardiomyopathy.

## Data Availability

The data used to support the findings of this study are available from the corresponding authors upon request.

## Supplementary Materials

Supplementary Materials Figure S1: glucose concentration, HbA1c level, TG level, ANP, and BNP gene expression increased after STZ injection. (A) Fast blood glucose was measured periodically from the control and diabetic mice in different time after STZ injection. (B) HbA1c level was detected 12 weeks after STZ injection. (C) Serum TG level was measured 12 weeks after STZ injection. (D) Myocardial ANP mRNA and BNP mRNA were quantified by real-time PCR 12 weeks after STZ injection. 18S gene was used as a housekeeping gene. ^∗∗^*P* < 0.01 significantly different from control. *n* = 6. Figure S2: I1PP1 is overexpressed by caudal vein injection of recombinant adenovirus carrying the I1PP1 genes in mice. 100 *μ*L recombinant adenovirus solution carrying I1PP1 gene (1 × 10^11^ PFU/mL) or vector was injected into the caudal vein of mice 8 weeks after STZ injection. I1PP1 and PP1 expression were quantified by western blot. GAPDH was used as a loading control. ^∗∗^*P* < 0.01 significantly different from WT-DCM without injection of recombinant adenovirus solution carrying I1PP1 gene; *^##^P* < 0.01 and #*P* < 0.05 significantly different from Ad-Vector+RIPK3^−/−^-DCM without injection of recombinant adenovirus solution carrying I1PP1 gene. *n* = 6.
