# Supplementary material for: RIPK3-Mediated Necroptosis in Diabetic Cardiomyopathy Requires CaMKII Activation
Source: Oxid Med Cell Longev. 2021 Jun 7;2021:6617816. doi: 10.1155/2021/6617816 (PMC8203407; doi:10.1155/2021/6617816)
Supplement: Supplementary Materials — Figure S1: glucose concentration, HbA1c level, TG level, ANP, and BNP gene expression increased after STZ injection. (A) Fast blood glucose was measured periodically from the control and diabetic mice in different time after STZ injection. (B) HbA1c level was detected 12 weeks after STZ injection. (C) Serum TG level was measured 12 weeks after STZ injection. (D) Myocardial ANP mRNA and BNP mRNA were quantified by real-time PCR 12 weeks after STZ injection. 18S gene was used as a housekeeping gene. ∗∗P < 0.01 significantly different from control. n = 6. Figure S2: I1PP1 is overexpressed by caudal vein injection of recombinant adenovirus carrying the I1PP1 genes in mice. 100 μL recombinant adenovirus solution carrying I1PP1 gene (1 × 10^11^ PFU/mL) or vector was injected into the caudal vein of mice 8 weeks after STZ injection. I1PP1 and PP1 expression were quantified by western blot. GAPDH was used as a loading control. ∗∗P < 0.01 significantly different from WT-DCM without injection of recombinant adenovirus solution carrying I1PP1 gene; ##P < 0.01 and #P < 0.05 significantly different from Ad-Vector+RIPK3−/−-DCM without injection of recombinant adenovirus solution carrying I1PP1 gene. n = 6. [file 6617816.f1.docx]

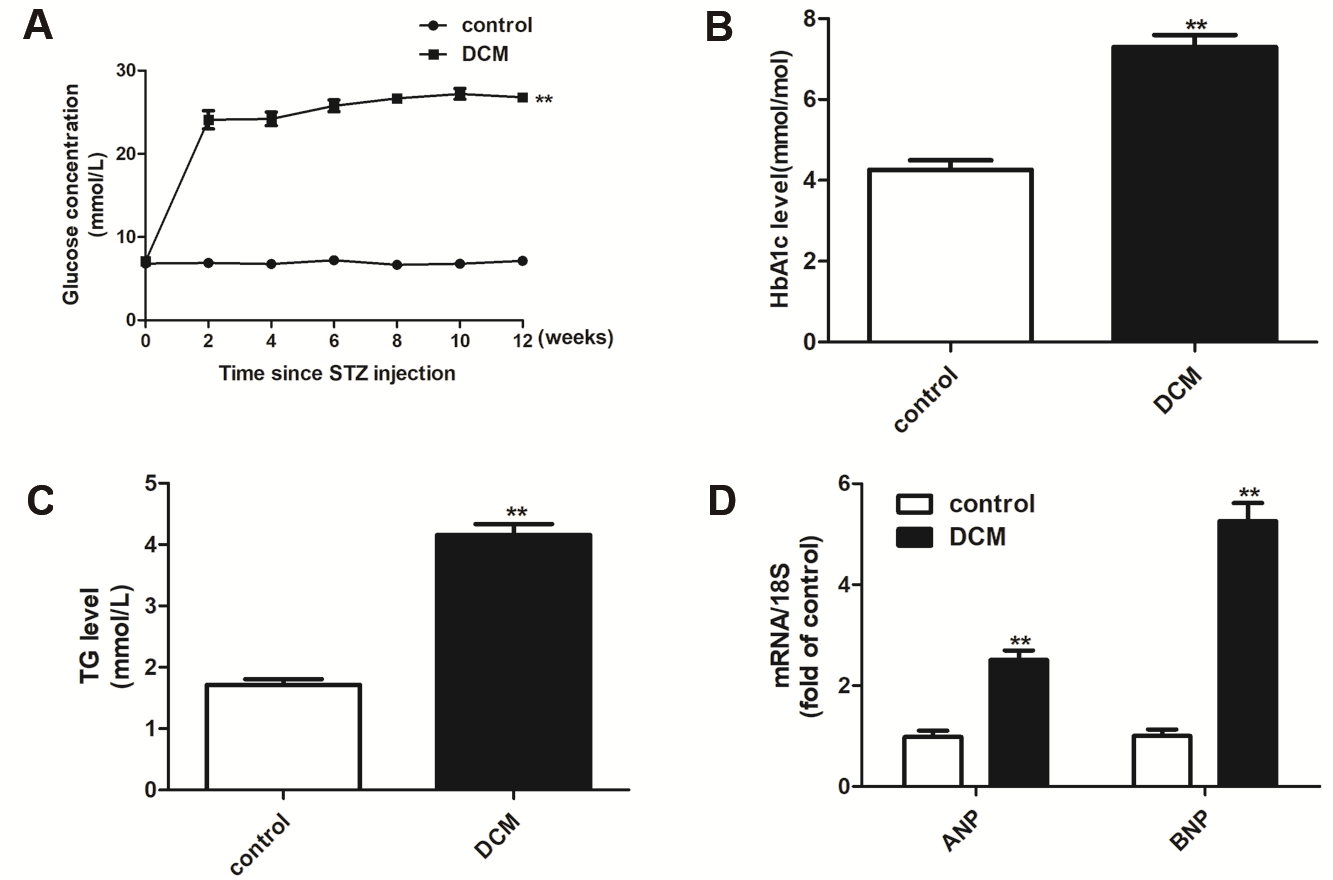


Figure S1 Glucose concentration, HbA1c level, TG level, ANP and BNP gene expression increased after STZ injection. (A) Fast blood glucose was measured periodically from the control and diabetic mice in different time after STZ injection. (B) HbA1c level was detected 12 weeks after STZ injection. (C) Serum TG level was measured 12 weeks after STZ injection. (D) Myocardial ANP mRNA and BNP mRNA were quantified by real-time PCR 12 weeks after STZ injection. 18S gene was used as a housekeep gene. ^**^*P* < 0.01 significantly different from control. n=6.


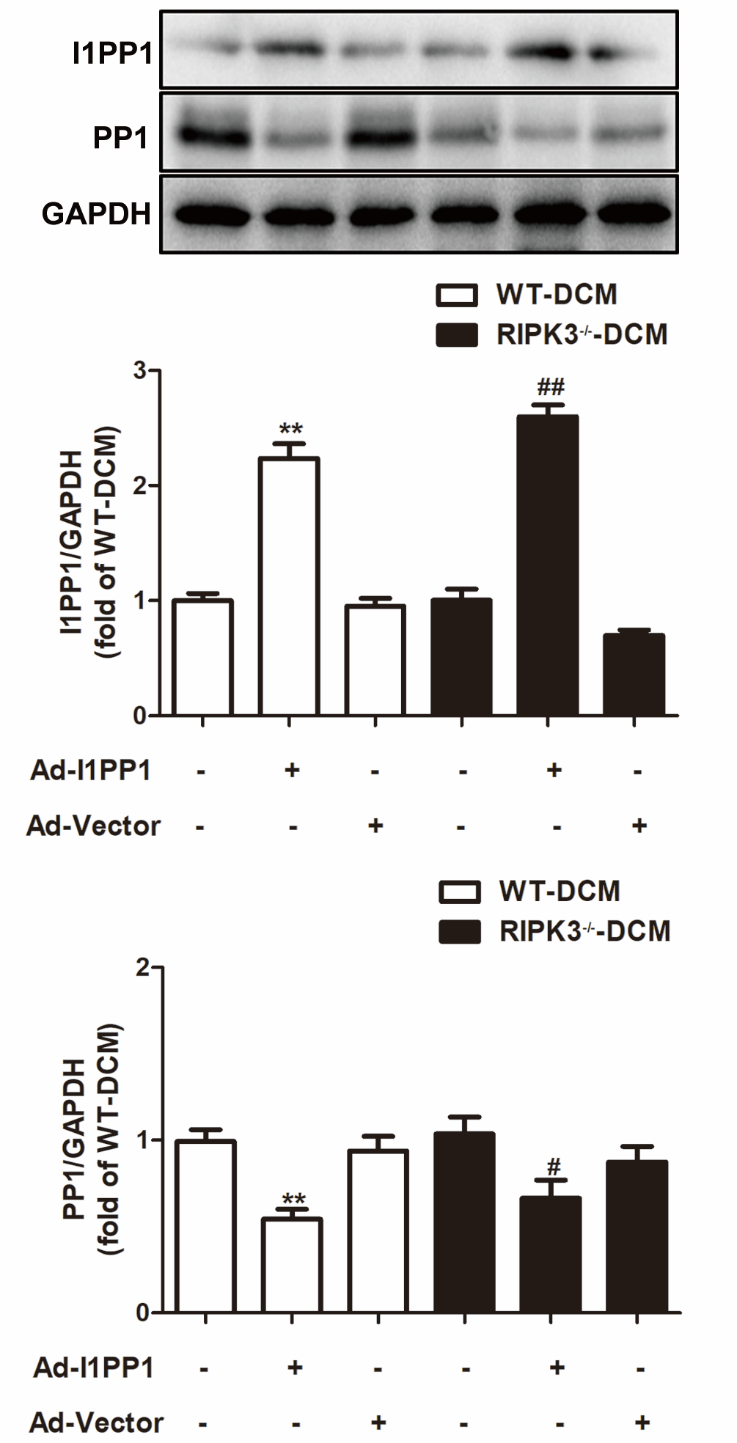


Figure S2 I1PP1 is over-expressed by caudal vein injection of recombinant adenovirus carrying the I1PP1 genes in mice. 100 μL Recombinant adenovirus solution carrying I1PP1 gene (1🞨10^11^ PFU/mL) or vector were injected into the caudal vein of mice 8 weeks after STZ injection. I1PP1 and PP1 expression were quantified by western blot. GAPDH was used as a loading control. ^**^*P* < 0.01 significantly different from WT-DCM without injection of recombinant adenovirus solution carrying I1PP1 gene; *^##^P* < 0.01, *^#^P* < 0.05 significantly different from Ad-Vector+RIPK3^-/-^-DCM without injection of recombinant adenovirus solution carrying I1PP1 gene. n=6.
